# Evaluation of Dimensional Changes in Maxillary and Frontal Sinus in Adult Patients With Anterior Open Bite and Normal Overbite: A Retrospective Cone Beam Computed Tomography (CBCT) Study

**DOI:** 10.7759/cureus.53710

**Published:** 2024-02-06

**Authors:** Sivasankari R S S, Nayeemullah Khan, Ratna Parameswaran, Srinivasan Boovaraghavan, Manini Nagi

**Affiliations:** 1 Orthodontics and Dentofacial Orthopaedics, Meenakshi Academy of Higher Education and Research, Chennai, IND

**Keywords:** cbct, distobuccal root of first molar, palatal root of first molar, pneumatization, maxillary sinus floor, frontal sinus, maxillary sinus

## Abstract

Introduction: An anterior open bite is a form of vertical discrepancy that presents as a lack of contact between maxillary and mandibular segments. The treatment modalities usually involve either intrusion of posterior teeth or extrusion of anterior or a combination of both. The anatomical relationship between the apex of the maxillary molar roots to the inferior wall of the maxillary sinus floor is crucial in planning posterior intrusion. The paranasal sinuses influence the growth of the facial structures that eventually get altered in various malocclusions. Studies have proven that the height of the sinus gets modified in anterior open bite owing to pneumatization. This study aims to evaluate the distance from the root apex of maxillary first molars (mesiobuccal, distobuccal, and palatal roots) to the maxillary sinus floor to evaluate the significance of the vertical pneumatization of the sinus on planning for true intrusion in anterior open bite and to assess the correlation between frontal and maxillary sinuses in an anterior open bite.

Methods: This retrospective study evaluated 30 pre-treatment cone beam computed tomographies (CBCTs) of patients out of which 15 were with anterior open bite and 15 with ideal overbite. Linear measurements were carried out using care stream software in CBCTs.

Results: There was a significant correlation between the distance of the palatal root and the distobuccal root of the maxillary first molar to the maxillary sinus floor bilaterally in the anterior open bite (p<0.04). A significant moderate positive correlation of the maxillary and frontal sinus height in anterior open bite (p<0.006). A significant moderate negative correlation between the distance from the palatal root to the maxillary sinus floor and maxillary sinus height in anterior open bite (p<0.001).

Conclusion: Vertical pneumatisation of the maxillary sinus has caused a significant negative correlation between the apex of the palatal root of the maxillary first molar tooth and the maxillary sinus floor in the anterior open bite. The palatal root being the closest to the sinus floor, and the distobuccal root being second nearest. There is a significant correlation between the height of the sinuses in the anterior open bite.

## Introduction

An anterior open bite is a form of vertical discrepancy that presents as a lack of contact between maxillary and mandibular segments. The skeletal features of an anterior open bite are commonly evident as tapered facial type, short ramus, and clockwise rotation of the mandible whereas the cephalometric feature depicts enlarged adenoids, decreased nasolabial angle, increased dentoalveolar height in the molar region, reduced dentoalveolar height in incisor region, steep mandibular plane angle and obtuse gonial angle. The soft tissue features include a long lower third of the face, incompetent lips, a narrow nose, and alar bases.

The paranasal sinuses influence the growth of the facial structures and eventually get altered in various malocclusions. Studies indicate that maxillary and frontal sinuses are enlarged in open bite cases. The major treatment modalities of the treatment involve either intrusion of posterior teeth or extrusion of anterior, or a combination of both. The anatomical relationship between the apex of the maxillary molar roots to the inferior wall of the maxillary sinus floor (MSF) is crucial for planning posterior intrusion. Since, the height of the maxillary sinus varies in different age groups and growth patterns, evaluation of root proximity, the thickness of the alveolar mucosa, and the height of the basal bone is essential to avoid sinus perforation and resorption of roots during intrusion of maxillary posterior teeth. Due to the shortcomings of 2D technologies such as superimposition of the transverse boundaries of the maxillary sinus and distortion and magnification of the images can be eliminated by using a 3D imaging technique [1]. In this study 3D imaging techniques have been utilized to overcome the shortcomings of 2D technologies.

The aim of this study is the bilateral evaluation of the basal bone height of all three roots (mesiobuccal, distobuccal, and palatal roots) of maxillary first molars, the transverse and vertical dimension of the maxillary sinus, the vertical dimension of the frontal sinus on patients with anterior open bite and normal overbite using a 3D imaging technique cone beam computed tomography (CBCT).

## Materials and methods

The study sample consists of 30 diagnostic CBCT records of patients (17 females and 11 males) who reported to our centre. Considering the 80% power of the study with an alpha error of 5%, the sample size calculation showed that the sample number in both groups was sufficient for attaining dependable results.

Inclusion criteria for the test and control groups

CBCT records of patients comprising males and females belonging to the age group of 18 to 27 years with an anterior open bite greater than 1 mm and an ideal overbite of about 2-3 mm were included in the test group. CBCT records of males and females belonging to the age group of 18 to 27 years with a normal overbite of about 2-3 mm and a normodivergent growth pattern were included in the control group.

Exclusion criteria

CBCT records of patients with permanent first molar tooth extraction, craniofacial deformities like cleft lip and palate, and congenitally missing, impacted, mutilated, or prosthetically replaced teeth were excluded from the study.

All the CBCT records were retrospectively analyzed. Out of which 15 records with anterior open bite and 15 records with normal overbite were taken. Due to beam-related and patient-related artifacts, two records with anterior open bite were excluded. CBCTs were taken using CBCT-KODAK 9500 (Carestream Health, Inc., NY, USA). CBCTs were exposed at 300 voxel size at 90KV, 10mA with a scanning time of 10 seconds. All the CBCTs were taken on this set of standards. CBCT images were taken in the patient’s upright position with the mid-sagittal plane perpendicular to the horizontal plane as recommended by the manufacturers. The acquired data were imported into the care stream (3D) software, product version 3.5.18.0. All CBCTs were viewed and measured by the same examiner to avoid any inter-observer error. This retrospective study protocol was reviewed and approved by the institutional review board (MADC/IRB-XXIII/2018/376).

CBCT image standardization

**Figure 1:**
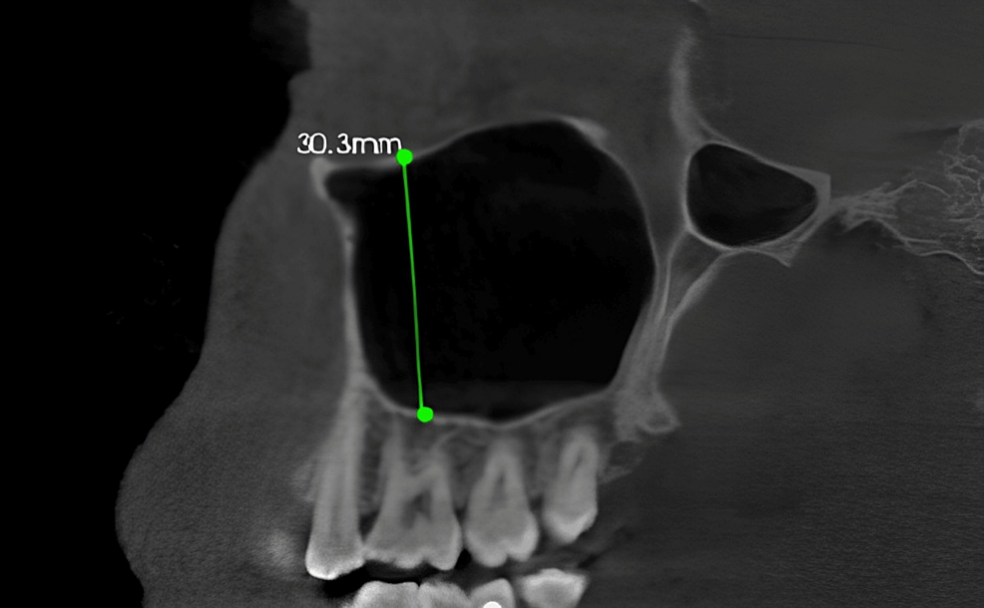
The linear measurement of the maxillary sinus height

The landmarks of the sinus were standardized by positioning the CBCT image such that the axial cut was equivalent to the maxillary posterior occlusal plane at the alveolar crest region, the sagittal cut was positioned between the middle of buccal and palatal cortices, and for the coronal cut, the axial image was positioned until the orientation axis was perpendicular to the buccal cortex. The linear measurement of the maxillary sinus height: It was measured bilaterally in the sagittal view from the lowest point of the orbital floor to the maxillary sinus floor at the first molar region in orthogonal slicing (Figure 1).

**Figure 2:**
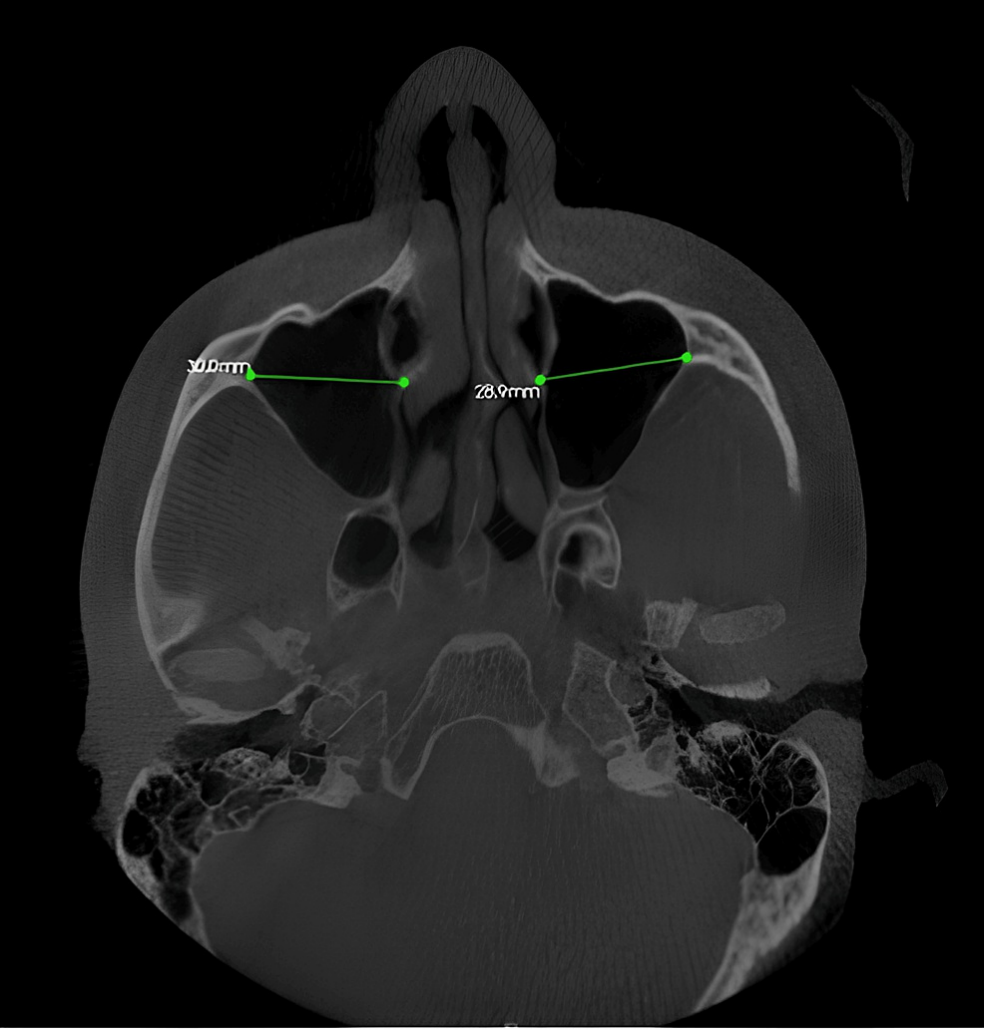
The linear measurement of the maxillary sinus widths

The linear measurement of the maxillary sinus width: The mediolateral width was obtained along the maximum dimension of the zygomatic region on both sides and was measured on the axial view in oblique slicing (Figure 2).

**Figure 3:**
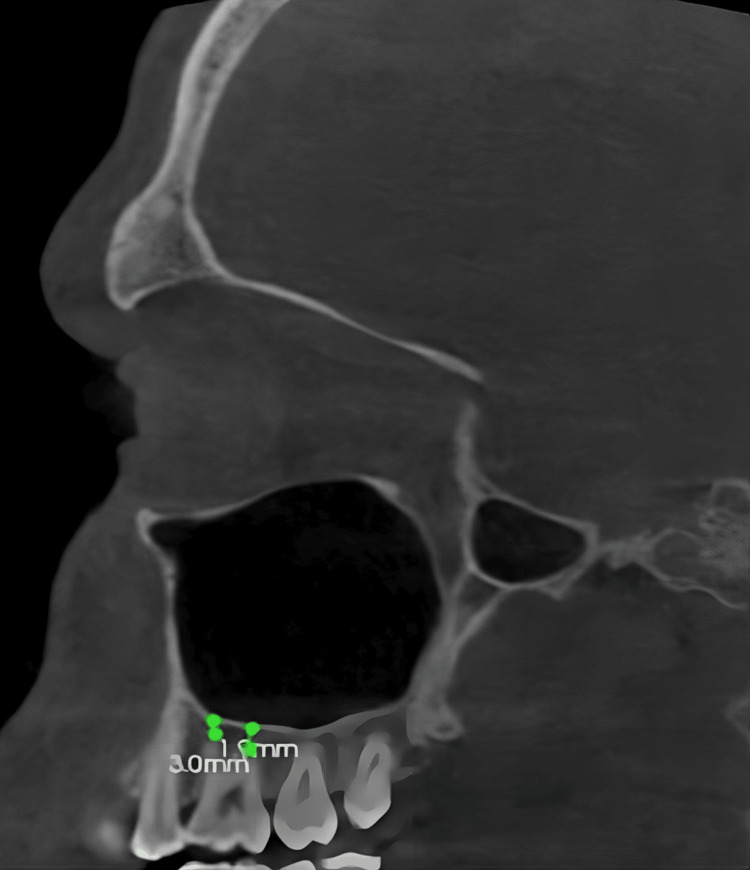
The linear measurements of the distance from mesiobuccal and distobuccal root apices to the sinus floor

The linear measurement of the basal bone height: The distance from mesiobuccal and distobuccal root apices of the maxillary first molar to the inferior-most point of the maxillary sinus floor on both sides were measured on the sagittal view in orthogonal slicing (Figure 3).

**Figure 4:**
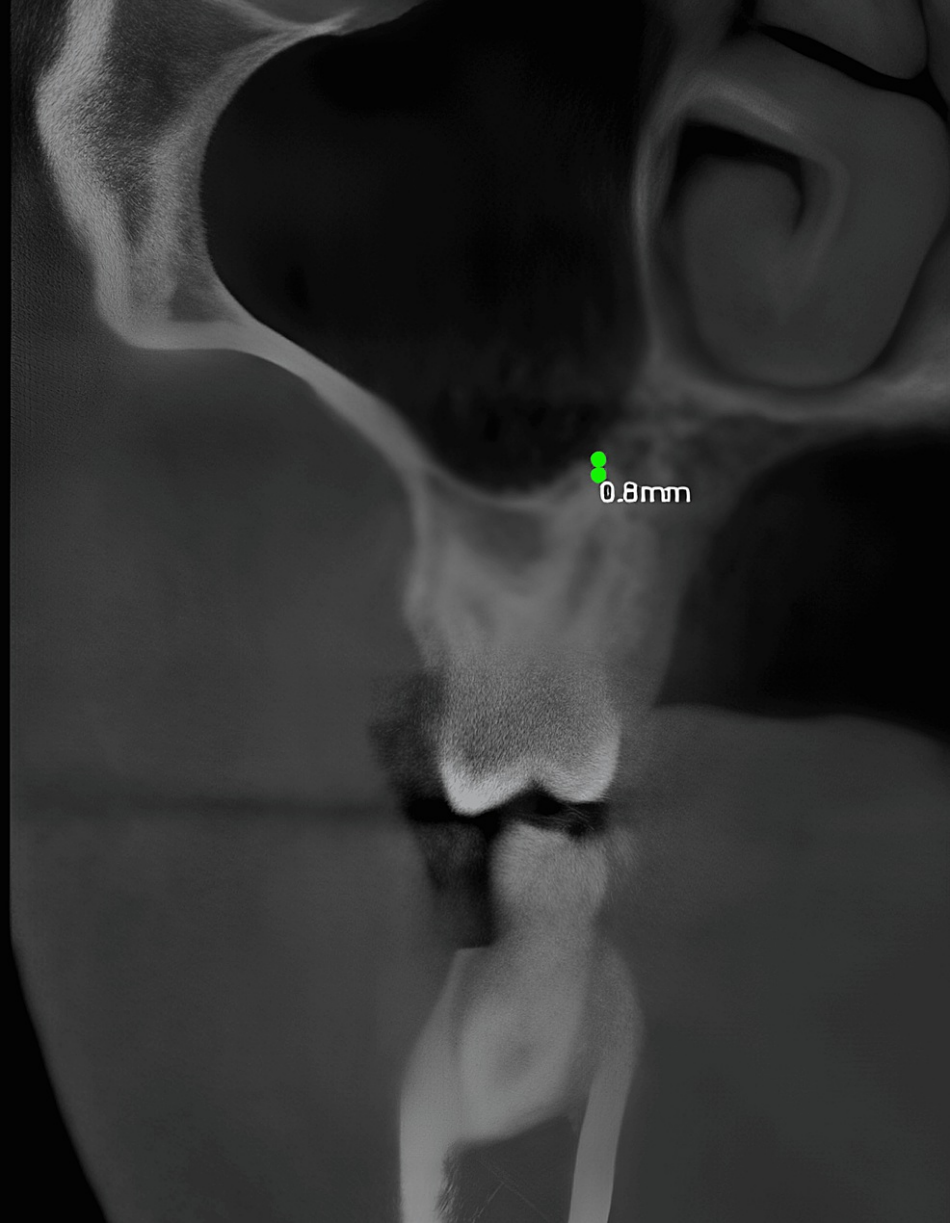
The linear measurement of the distance from the palatal root apex to the sinus floor

The distance from the palatal root apex of the maxillary first molar to the inferior lining of the maxillary sinus floor on both sides was measured on the coronal view in curved slicing (Figure 4).

**Figure 5:**
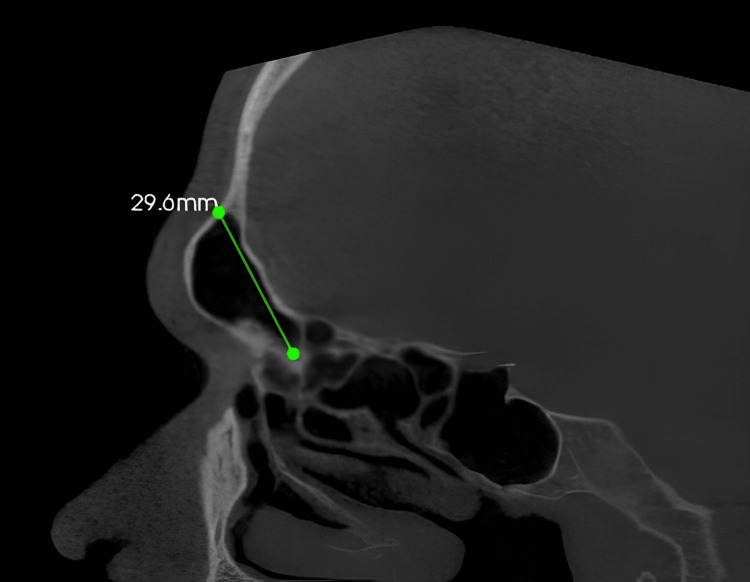
The linear measurement of the frontal sinus height

The linear measurement of the frontal sinus height: The distance from the inferior portion of the frontal bone to the floor of the orbital portion of the frontal bone is measured on the sagittal view in oblique slicing (Figure 5). 

## Results

The above-mentioned parameters were analyzed using the SPSS software, version 20.0 (IBM Corp., Armonk, NY). Quantitative data were represented as Mean and SD (distance from root apex to the maxillary sinus floor, maxillary sinus width, and height). Kolmogrov-Smirnov test with a p-value of <0.05, which proved the data was non-normal. Student’s t-test (parametric test)/Mann Whitney U test (the non-parametric equivalent of student’s t-test) was used for inter-group comparison (difference in radiological parameters between control and study groups). A p-value of <0.05 was considered statistically significant in the current study.

There was a significant difference in the measured parameters for the distobuccal and palatal roots. There was no significant difference in the Mesiobuccal root between the groups (Table 1).

**Table 1:** Inter-group comparison of distance from molar roots apices to the floor of the maxillary sinus p<0.05 is considered significant; N: Mean; SD: Standard Deviation.

Variable	Group	N	SD	t Statistic	P-value
MB – right	Control	1.21	1.97443	0.07	0.94
	Study	1.26	1.35636		
MB – Left	Control	1.67	2.16817	0.26	0.79
	Study	1.49	1.42522		
DB – right	Control	2.03	0.75939	2.2	0.031*
	Study	1.35	0.87331		
DB – Left	Control	2.13	1.08527	2.1	0.041*
	Study	1.35	0.89751		
Palatal – right	Control	2.18	1.6389	2.2	0.036*
	Study	0.98	1.32568		
Palatal – Left	Control	2.51	2.18987	2.1	0.045*
	Study	1.01	1.71108		

There was no significant difference between the maxillary sinus width and height (Table 2).

**Table 2:** Inter-group comparison of maxillary sinus width and height on both right and left side p<0.05 is considered significant; N: Mean; SD: Standard Deviation.

Variable	Group	N	SD	t statistic	P-value
Maxillary sinus width - Right	Control	26.82	4.96908	0.77	0.44
	Study	28.01	3.25749		
Maxillary sinus width - Left	Control	27.12	5.73737	0.08	0.93
	Study	27.29	4.6451		
Maxillary sinus height - Right	Control	36.95	4.03642	0.24	0.81
	Study	36.61	3.61237		
Maxillary sinus height - Left	Control	37.01	3.96122	0.82	0.41
	Study	38.07	2.99868		

The parameters measured between the maxillary sinus width and height on both the right and left sides of both groups did not show statistical significance (Table 3).

**Table 3:** Intragroup comparison of maxillary sinus width and height between the left and right sides of both groups p<0.05 is considered significant; N: Mean; SD: Standard Deviation.

Variable	Group	N	SD	t statistic	P-value
Maxillary sinus width - Control	Right	26.82	4.96908	0.15	0.87
	Left	27.12	5.73737		
Maxillary sinus height – Control	Right	36.9467	4.03642	0.04	0.96
	Left	37.0133	3.96122		
Maxillary sinus width - Study	Right	28.0133	3.25749	0.49	0.62
	Left	27.2867	4.6451		
Maxillary sinus height - Study	Right	36.6067	3.61237	0.37	0.23
	Left	38.0733	2.99868		

**Figure 6:**
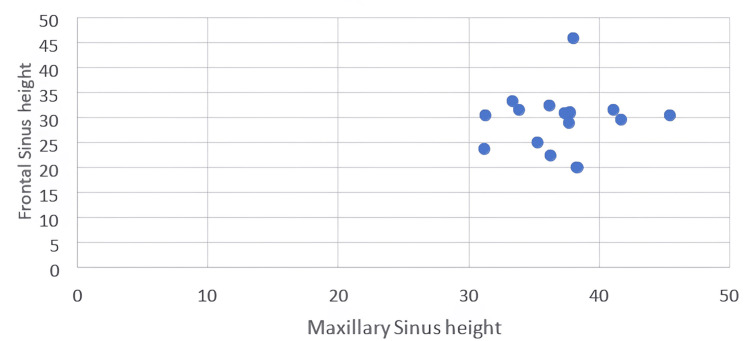
Scatter plot showing the linear relationship between the height of the maxillary sinus and frontal sinus in the control group

**Figure 7:**
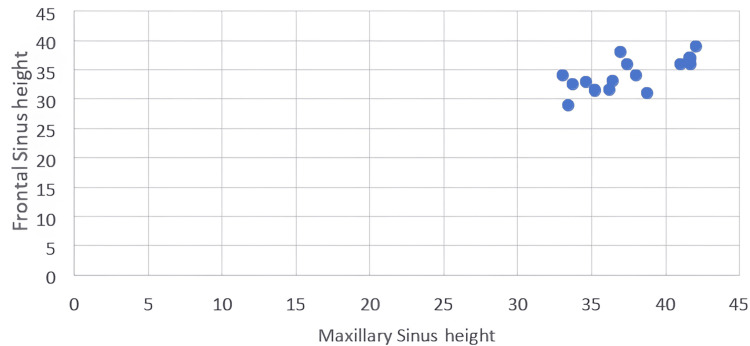
Scatter plot showing the linear relationship between the height of the maxillary sinus and frontal sinus in the study group

Significant moderate positive correlation between frontal sinus height and maxillary sinus height in the study group alone (Table 4) (Figures 6, 7).

**Table 4:** Bivariate correlations between the frontal sinus and maxillary sinus p<0.05 is considered significant; r: Pearson’s correlation coefficient.

Variables compared	r	Strength of correlation	Significance (P-value)
Frontal sinus height vs Maxillary sinus height (Control)	0.4	Moderate positive	Non- significant (0.13)
Frontal sinus height vs Maxillary sinus height (Study)	0.674	Moderate Positive	Significant (0.006)**

Significant moderate negative correlation between distance from the palatal root to the maxillary sinus floor and maxillary sinus height, in the study group alone. As the distance from the palatal root decreases on the left side, there is a significant increase in maxillary sinus height (Table 5).

**Table 5:** Bivariate correlations between distance from palatal root and maxillary sinus height (left side) p<0.05 is considered significant; r: Pearson’s correlation coefficient.

Variables compared	r	Strength of correlation	Significance (P-value)
Distance from palatal root vs Maxillary sinus height (Control)	-0.335	Weak negative	Non- significant (0.22)
Distance from palatal root vs Maxillary sinus height (Study)	-0.748	Moderate negative	Highly Significant (0.001)**

Significant moderate negative correlation between distance from the palatal root to the maxillary sinus floor and maxillary sinus height, in the study group alone. As the distance from the palatal root decreases on the right side, there is a significant increase in maxillary sinus height (Table 6).

**Table 6:** Bivariate correlations between distance from palatal root and maxillary sinus height (right side) p<0.05 is considered significant; r: Pearson’s correlation coefficient.

Variables compared	r	Strength of correlation	Significance (P-value)
Distance from palatal root vs Maxillary sinus height (Control)	-0.429	Weak negative	Non- significant (0.11)
Distance from palatal root vs Maxillary sinus height (Study)	-0.644	Moderate negative	Highly Significant (0.009)**

## Discussion

Anterior open bite manifests as an absence of contact between the opposing teeth. The severity of anterior open bite is dependent on the aetiology. Despite extensive available literature, the rationale behind the treatment of anterior open bite has posed a challenge in the field of orthodontics.

The architecture of the cranium is harmonious and dynamically responds to mechanical stresses. The paranasal sinuses are air-filled areas adjoining the bones of the maxilla, frontal, sphenoid, and ethmoid. The maxillary sinus has a huge impact on the growth and development of craniofacial structures. Once the sinus cavity develops within the maxillary bone, it gets occupied by air and this physiological process is called pneumatization. The expansion of the maxillary sinus into the adjacent structures due to pneumatization still lacks concrete literature evidence. 

Treating anterior open bite is arduous because true intrusion of posteriors is the most viable option. Since the maxillary posterior teeth are in juxtaposition with the floor of the maxillary sinus, a cautious evaluation must be done to prevent the sinus floor from breaching. Though various diagnostic aids help in the study of proximation of the posterior teeth to the maxillary sinus floor using 2D technologies, since the maxillary sinus is a three-dimensional structure, it would be more reliable to depend on CBCT.

One of our objectives was to assess the topographic association between the maxillary sinus floor and the first molar roots. The topographic anatomy of the maxillary sinus floor and the roots of the molar teeth were classified into five types [2] (Table 7).

**Table 7:** Classification of topographic anatomy of the maxillary sinus floor and the roots of the molar teeth

TYPE	DESCRIPTION
TYPE I	a wide range of distances from the root apices of buccal and palatal roots to the maxillary sinus floor.
TYPE II	reduced gap and the root apices are exactly below the floor of the maxillary sinus.
TYPE III	perforation of the buccal root apex into the floor of the maxillary sinus.
TYPE IV	an invasion of the palatal root apex to the maxillary sinus floor.
TYPE V	penetration of buccal and palatal root apices into the inferior wall of the maxillary sinus floor.

Numerous studies have been conducted by various authors to evaluate the proximity of second molars to the maxillary sinus. The apex of the mesiobuccal root of the second molar was near the inferior wall of the maxillary sinus floor but the mucosa was thinnest at the distobuccal root apex [2-5].

It was found that on a hyperdivergent facial growth pattern, the distance between the palatal root and maxillary sinus floor was decreased. The distobuccal root was second closest to the maxillary sinus floor for the first molars bilaterally. This was in concordance with the studies [6-10] that evaluated the significance of facial biotype in association with the location of the root apices of the maxillary second molar to the maxillary sinus floor. They found that the second molar roots are closer to the maxillary sinus floor in normodivergent and hyperdivergent facial patterns. It was the farthest in hypodivergent patterns. The basal bone height was also reduced in the patients with an anterior open bite on hyperdivergent growth patterns. The palatal root projection of the maxillary first molar along the maxillary sinus cavity was common and the mesiobuccal root of the second molar was second closest to the sinus floor [11].

On comparison of the topographic relation of the maxillary sinus to the roots of the posterior teeth using OPG and CT, results showed that the majority of the roots projected into the maxillary sinus in OPG, whereas no actual projection was observed in CT for the same records [12].

In anterior open bite cases, It is found that pneumatization of the maxillary sinus occurs. As a result, the height of the maxillary sinus increases and the floor descends. This was confirmed in our study as we obtained a significant negative correlation between the maxillary sinus height and the first molar palatal root distance bilaterally. This may pose a problem when molars need to be intruded.

The true intrusion of molars is one of the main treatment modalities for anterior open bite correction. This can be achieved by various means, such as temporary anchorage devices (TADs), vertical holding appliances, bite blocks, and rapid molar intrusion (RMI) devices [13]. The amount of molar intrusion depends on the extent of the anterior open bite; 1 mm of intrusion corrects 2 mm of anterior open bite [13]. Hence, the distance between the molar roots and the sinus floor must be carefully calculated before the intrusion. If the space is sufficient, true intrusion can be achieved without any untoward effect. But when space is reduced, and the molar roots breach or touch the sinus floor, resorption may occur. Another unwarranted effect may be the buccal flaring of the molars. This can be negated using a transpalatal arch. Hence, our study plays an essential role in the estimation of the vertical distance from the first molar roots to the inferior wall of the maxillary sinus.

In our study, we compared the height of maxillary and frontal sinuses, and a significant moderate positive correlation was obtained. This suggests that vertical pneumatization of the sinuses is also occurring along with the vertical discrepancy. Many authors assessed frontal and maxillary sinuses in different types of skeletal malocclusions. A significant correlation was found between the frontal sinus and skeletal malocclusion (p < 0.05) compared to the maxillary sinus. Hence, frontal sinus dimensions can be used for the prediction of skeletal growth patterns. It has been noted that in hyperdivergent cases, the frontal sinus is found to be elongated. This is because of the poor transmission of occlusal forces along the trajectories of nasal pillars and reduced muscular activity. The increased frontal sinus size was associated with a reduced inclination of the anterior cranial base, increased anterior facial height, and gonial angle [14-16]. Goymen et al. [17] could not find any relevance between the growth pattern and the size of the sinuses.

Tooth loss, number, and location of roots affect the pneumatization of the maxillary sinus [6]. Schriber et al. found that the vertical bone height that is altered after tooth loss in the posterior maxilla is mainly due to resorption of the alveolar crest and not due to pneumatization of the maxillary sinus [18].

Study limitations

The skeletal open bite has not been taken into consideration in this study. The sample size could be increased for generalisation. Further studies can be carried out to estimate the treatment results after the intrusion of the posterior teeth and also evaluate the stability after the treatment of the anterior open bite.

## Conclusions

A significant correlation was found between the distance of the palatal and distobuccal root of the maxillary first molar to the maxillary sinus floor bilaterally in the anterior open bite. A significant moderate positive correlation of the maxillary and frontal sinus height in anterior open bite was found. A significant moderate negative correlation was observed between the distance from the palatal root to the maxillary sinus floor and maxillary sinus height in the anterior open bite. However, no correlation was seen for the mesiobuccal root of the first molar tooth bilaterally in the anterior open bite and no correlation was observed for the transverse dimensions of the maxillary sinus width bilaterally in the anterior open bite.
